# Application of Multivariate Adaptive Regression Splines to Estimate Fatty Liver Index in Healthy Young Taiwanese Men

**DOI:** 10.3390/diagnostics16050795

**Published:** 2026-03-07

**Authors:** Po-Chung Chen, Chung-Chi Yang, Dee Pei, Ta-Wei Chu, Jyh-Gang Leu

**Affiliations:** 1 Division of Family Medicine, Taoyuan Armed Forces General Hospital, Taoyuan 325208, Taiwan; tarsal0925@gmail.com; 2 Division of Cardiovascular Medicine, Taoyuan Armed Forces General Hospital, Taoyuan 325208, Taiwan; t220979@gmail.com; 3 Cardiovascular Division, Tri-Service General Hospital, National Defense Medical University, Taipei 114202, Taiwan; 4 School of Medicine, National Tsing Hua University, Hsinchu 300044, Taiwan; 5 Institute of Bioinformatics and Structural Biology, National Tsing Hua University, Hsinchu 300044, Taiwan; 6 Division of Endocrinology and Metabolism, Department of Internal Medicine, Fu Jen Catholic University Hospital, College of Medicine, Fu Jen Catholic University, New Taipei 242062, Taiwan; peidee@gmail.com; 7 Department of Obstetrics and Gynecology, Tri-Service General Hospital, National Defense Medical University, Taipei 114202, Taiwan; taweichu@gmail.com; 8 MJ Health Research Foundation, Taipei 114066, Taiwan; 9 Division of Nephrology, Department of Internal Medicine, Shin Kong Wu Ho-Su Memorial Hospital, Taipei 111045, Taiwan; 10 School of Medicine, Fu-Jen Catholic University, New Taipei 242062, Taiwan

**Keywords:** multivariate adaptive regression spline, fatty liver index, young, Taiwanese young men

## Abstract

**Background**: Non-alcoholic fatty liver disease (NAFLD) represents the most widespread chronic liver disorder globally, impacting roughly 30% of the general population. Numerous factors have been linked to NAFLD, including obesity, type 2 diabetes, diet, physical inactivity, age, sex, genetic factors, and metabolic syndrome. Previous research predominantly treated NAFLD as a categorical outcome, providing less granular data compared to the continuous fatty liver index (FLI). This investigation enrolled healthy young Taiwanese men and applied multivariate adaptive regression spline (MARS) modeling to develop a predictive equation. Our aims were twofold: 1. To assess the predictive accuracy of traditional multiple linear regression (MLR) versus MARS. 2. To construct a MARS-derived equation for estimating FLI in this demographic. **Methods**: Data originated from the Taiwan MJ Cohort, comprising 5496 men aged 20–50 years not using medications for metabolic syndrome. MARS was used to formulate the FLI estimation equation. Model performance was compared using symmetric mean absolute percentage error (SMAPE), relative absolute error (RAE), root relative squared error (RRSE), and root mean squared error (RMSE). **Results**: Evaluation indicated that MARS yielded lower estimation errors than MLR, demonstrating its superior performance. The derived equation is: FLI = 65.224 − 0.436 × B1 − 0.490 × B2 + 0.252 × B3 − 2.962 × B4 + 2.231 × B5 − 0.292 × B6 + 0.189 × B7 − 0.361 × B8 − 0.699 × B9 + 0.160 × B10 − 2.715 × B11 + 0.799 × B12 − 0.153 × B13 + 0.084 × B14 − 35.274 × B15 − 4.424 × B16. **Conclusions**: Using MLR as a benchmark, our analysis revealed that MARS delivered better predictive performance. The presented equation explains 62.7% of the variance in FLI (r^2^ = 0.627). Based on standardized variable importance scores (nsubsets metric), CRP emerged as the most influential predictor, followed by WBC, UA, HDL-C, AST, age, ALT, FPG, SBP, and LDL in this cohort of healthy young Taiwanese men.

## 1. Introduction

Non-alcoholic fatty liver disease (NAFLD) is the most prevalent chronic liver condition globally, affecting approximately 30% of the general population [1]. Once considered a benign hepatic manifestation of obesity, NAFLD is now recognized as a multisystem disorder closely intertwined with insulin resistance, type 2 diabetes, dyslipidemia, and cardiovascular disease [2,3,4]. Its clinical spectrum ranges from simple steatosis to non-alcoholic steatohepatitis (NASH), fibrosis, cirrhosis, and even hepatocellular carcinoma [5]. Given its strong association with metabolic dysfunction, an international consensus panel recently proposed redefining NAFLD as metabolic dysfunction-associated fatty liver disease (MAFLD) [6]. This paradigm shift emphasizes positive diagnostic criteria based on the presence of metabolic risk factors rather than the exclusion of other liver diseases, thereby improving clinical relevance and inclusivity [7].

Epidemiological studies have consistently identified key risk factors for fatty liver, including central obesity, hypertriglyceridemia, low high-density lipoprotein cholesterol (HDL-C), elevated fasting plasma glucose, hypertension, sedentary behavior, and genetic predisposition [8,9,10]. However, a substantial proportion of existing research treats NAFLD as a binary outcome—either present or absent—typically diagnosed via imaging or biomarkers with predefined cutoffs. While this approach facilitates case-control comparisons, it discards valuable information about the degree of hepatic fat accumulation and limits the ability to detect dose–response relationships or subtle metabolic gradients [11].

To address this limitation, the fatty liver index (FLI) was developed as a continuous, noninvasive surrogate for hepatic steatosis, combining body mass index (BMI), waist circumference, serum triglycerides, and γ-glutamyltransferase (γ-GT) into a single score ranging from 0 to 100 [12]. FLI has been validated against ultrasound and magnetic resonance imaging in multiple populations and demonstrates strong predictive performance for incident type 2 diabetes and cardiovascular events [13,14]. By modeling FLI as a continuous outcome, researchers can uncover more nuanced associations between metabolic, inflammatory, and lifestyle variables and the severity of fatty liver, even in ostensibly healthy individuals.

In parallel, advances in machine learning have opened new avenues for modeling complex biomedical relationships. Among these, multivariate adaptive regression splines (MARS) offer a unique balance between flexibility and interpretability [15]. Unlike “black-box” algorithms such as deep neural networks, MARS constructs a piecewise linear model using hinge functions that automatically detect nonlinearities and interactions without requiring prespecified functional forms [16]. The resulting model can be expressed as a transparent mathematical equation—making it particularly suitable for clinical translation and hypothesis generation. MARS has demonstrated superior predictive accuracy over traditional multiple linear regression (MLR) in diverse domains, including livestock weight estimation [17] and cardiovascular risk prediction [18], yet its application in hepatology remains scarce.

Notably, because BMI, waist circumference, triglycerides, and γ-GT are already embedded in the FLI formula, including them as predictors would introduce circularity and inflate model performance. Therefore, in this study, we deliberately excluded these four variables to explore independent determinants of FLI—such as inflammatory markers (e.g., C-reactive protein, white blood cell count), liver enzymes, renal function, and lifestyle factors—in a cohort of healthy young Taiwanese men aged 20–50 years who were free of medications for metabolic conditions. This population is of particular interest because early metabolic perturbations may manifest before the onset of overt obesity or diabetes, offering a window into the initial drivers of fatty liver development.

The principal goals of this research were:

(1) To compare the predictive accuracy of MARS against traditional MLR in estimating FLI, using robust error metrics;
(2) To derive an interpretable MARS-based equation that ranks the relative importance of non-FLI variables in predicting hepatic steatosis risk in this understudied demographic.

While the primary aim of this study is predictive—to develop an accurate model for estimating FLI—the use of MARS is specifically intended to provide interpretable, mechanistic insight into the factors driving hepatic steatosis risk, beyond the components of the FLI itself. By modeling FLI as a continuous variable, this approach enables fine-grained risk stratification, identifying individuals across a spectrum of risk rather than a binary NAFLD classification. The resulting equation is designed to be practically implementable using common clinical variables, potentially aiding in early screening and personalized preventive strategies in primary care or health check-up settings by highlighting modifiable risk factors such as inflammation, dyslipidemia, and hyperglycemia.

## 2. Materials and Methods

### 2.1. Participant and Study Design

The data used in this investigation were derived from the Taiwan MJ Cohort, an established, ongoing prospective cohort encompassing health examinations performed by the MJ Health Screening Centers in Taiwan. This comprehensive dataset includes over 100 essential biological indicators, including anthropometric measurements, blood biochemical analyses, and imaging results. Participants also provided information on personal and family medical history, current health status, lifestyle, physical exercise, sleep patterns, and dietary habits via a self-administered questionnaire.

Study data were acquired from the MJ Clinic Database, maintained by the MJ Health Research Foundation. A general consent for future anonymous research was obtained from participants at the time of their original health check-up. The use of this data was authorized by the MJ Health Research Foundation (Authorization No.: MJHRF2024002A). As this is a secondary database analysis not involving new sample collection, a project-specific consent form was not required. Detailed procedures for the initial data collection are available in the annual technical report published by the MJ Health Research Foundation [19].

This study was reviewed and approved by the Institutional Review Board of the Tri-Service General Hospital (IRB No.: A202405006), receiving an expedited review due to its nature as a secondary analysis.

**Figure 1:**
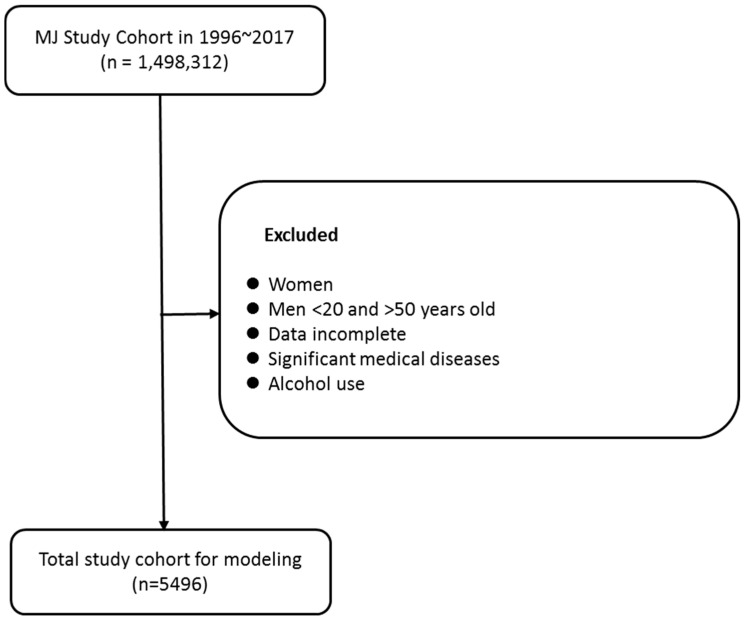
Participants’ selection process.

The initial enrollment for the cohort included 1,498,312 subjects. Following the application of our predefined exclusion criteria, 5496 male subjects were retained for the final analysis (as detailed in the flow chart, Figure 1).

Inclusion Criteria:

1. Men between 20 and 50 years old
2. No history of significant medical diseases such as stroke, myocardial infarction, or heart failure
3. No medications for metabolic syndrome
4. Without alcohol consumption

Subjects aged from 20 to 50 years were selected to capture individuals in the preclinical and early metabolic dysfunction phase, prior to the development of overt cardiometabolic diseases [20,21]. This design facilitates the study of incipient risk factors for fatty liver. The sample was restricted to men to avoid the substantial confounding effects of female sex hormones (e.g., estrogen) on liver fat accumulation, lipid profiles, and inflammatory markers—factors that differ by menopausal status, contraceptive use, and menstrual cycle phase [22,23]. This approach ensures cohort homogeneity and enhances model interpretability by removing these complex, sex-specific variables.

### 2.2. Measurements and Biochemical Analysis

On the day of the health examination, trained personnel, typically a senior nurse, documented participants’ personal history details, including current and past habits such as tobacco, alcohol, and betel nut consumption, as well as their education level. Body weight (kg) was accurately recorded using a calibrated electronic scale. Both systolic blood pressure (SBP) and diastolic blood pressure (DBP) were measured using a standardized electric sphygmomanometer.

Blood samples were collected following a minimum 10 h fasting period. The plasma was promptly separated from the whole blood within one hour of collection and subsequently stored at degrees Celsius until lipid profile testing. Lipid profile analysis was performed as follows: Total cholesterol (TC) and triglyceride (TG) concentrations were determined using a dry, multi-layer analytical slide method with the Fuji Dri-Chem 3000 analyzer (Fuji Photo Film, Tokyo, Japan). High-density lipoprotein cholesterol (HDL-C) and low-density lipoprotein cholesterol (LDL-C) concentrations were quantified using an enzymatic cholesterol assay method subsequent to dextran sulfate precipitation. Further details on the methodology and standardized procedures may be found in our previous related work [24].

### 2.3. Traditional Statistics

Data are presented as the means ± standard deviations. To evaluate differences in continuous variables between groups, specific statistical tests were used based on the nature of the compared variables:

1. T-tests were used to assess the difference in means between two independent groups, specifically between married and unmarried participants.
2. Analysis of Variance (ANOVA) was applied when comparing differences across groups categorized by ordinal variables, such as education and income levels.
3. Pearson correlation coefficient was calculated to analyze the linear relationships between all continuous variables and the primary outcome measure, the FLI.

Furthermore, Multiple Linear Regression (MLR) was performed to serve as a benchmark for comparison against the performance of the various machine learning models. All statistical assessments were two-sided, and a *p*-value less than 0.05 (*p* < 0.05) was defined as the threshold for statistical significance. All data analyses were executed using SPSS 10.0 for Windows (SPSS, Chicago, IL, USA).

### 2.4. Description of the Study Data

Table 1 defines the 30 clinical variables used in this study. We gathered the following dependent variables from our study participants:, white blood cell (WBC) count, hemoglobin level, platelet count, total bilirubin (TBIL), total protein, albumin, globulin, aspartate aminotransferase (AST), alanine aminotransferase (ALT), gamma-glutamyltransferase (γ-GT), lactate dehydrogenase (LDH), creatinine, uric acid, TG, HDL-C, LDL-C, thyroid-stimulating hormone (TSH) level, C-reactive protein (CRP) level, educational level, marriage status, sleep time, and SBP and DBP level. The sleep time was an ordinal variable as shown in Table 1. Finally, the equation of FLI = ey/(1 + ey) × 100, where y = 0.953 × ln(triglycerides, mg/dL) + 0.139 × (BMI kg/m^2^) + 0.718 × ln(γ-GT, U/L) + 0.053 × (waist circumference, cm) − 15.745 [25]. Since BMI, waist circumference, γ-GT, and triglyceride were used in the calculation of FLI, they were excluded in the MARS.

### 2.5. Machine Learning Analysis: MARS and Model Evaluation

The dataset was investigated using the MARS technique, a powerful, non-parametric modeling approach well-suited for high-dimensional data, capable of crafting adaptable models. The methodology uses an expansion structure based on product spline basis functions. Crucially, the model is built autonomously through data-driven mechanisms [26]; this includes determining the number of basis functions, the attributes associated with each function (e.g., product degree), and the placement of knots. This strategy is inspired by recursive partitioning principles, similar to methods like Classification and Regression Trees (CART), allowing MARS to effectively capture complex higher-order interactions.

#### 2.5.1. Model Training and Validation

Data Partitioning

For the analysis, the dataset was initially divided into two segments: an 80% training set used for model construction and a separate 20% testing set designated for final model assessment.

Hyperparameter Tuning

During the training phase, MARS models require the tuning of specific hyperparameters to ensure optimal performance. To achieve this, the 80% training dataset was further divided into two random segments: one for model formulation using a distinct set of hyperparameters, and the other for validation. A comprehensive grid search approach was implemented, systematically exploring all possible combinations of hyperparameters to identify the best configuration.

Benchmark Comparison

To establish a comparative context, the averaged performance metrics derived from the tuned MARS model were used to contrast its performance with that of the MLR model, which served as the benchmark. Both the MARS models and the MLR model were trained and tested using the exact same data partitions.

#### 2.5.2. Model Evaluation: Performance Metrics

The predictive effectiveness of the MARS model was evaluated using the 20% testing dataset. Since the target variable in this study is a continuous, numerical parameter, the chosen evaluation metrics to compare model performance included:

Symmetric Mean Absolute Percentage Error (SMAPE)
Root Relative Squared Error (RRSE)
Root Mean Squared Error (RMSE)

SMAPE was calculated as: SMAPE = (100%/n) × Σ [|Actual − Predicted|/((|Actual| + |Predicted|)/2)], where n is the number of observations. This metric was selected for its robustness when dealing with values near zero, as it avoids the denominator instability of standard MAPE.

The model configuration that exhibited the lowest Root Mean Squared Error (RMSE) when applied to the validation dataset was selected as the optimal MARS model. This optimal MARS model was then compared against the benchmark MLR model using the testing dataset. The specific values for these metrics can be found in Table 2.

All methods were performed using R software version 4.0.5 and RStudio version 1.1.453 with the required packages installed [27,28]. The implementations of MARS were the “earth” R package version 5.3.3 [29] with “caret” R package version 6.0–94 [30]. The MLR was implemented using the “stats” R package version 4.0.5, and the default setting was used to construct the models.

## 3. Results

### Demographic Characteristics

A total of 5496 healthy young Taiwanese men aged 20–50 years were included in the final analysis. Participant characteristics are summarized in Table 3. The mean age was 36.8 ± 7.8 years, and the mean FLI was 32.6 ± 26.7. Most participants were married (56.8%) and had attained at least a university-level education (77.3%). Significant differences in income and sleep duration were observed between subgroups (all *p* < 0.001), whereas educational attainment showed no significant association with FLI in preliminary comparisons.

Table 4 presents Pearson correlation coefficients between FLI and continuous biochemical and demographic variables. FLI showed significant positive correlations with age (r = 0.172), systolic blood pressure (SBP; r = 0.344), diastolic blood pressure (DBP; r = 0.344), white blood cell count (WBC; r = 0.354), hemoglobin (r = 0.193), platelets (r = 0.205), fasting plasma glucose (FPG; r = 0.278), globulin (r = 0.171), alkaline phosphatase (ALP; r = 0.156), aspartate aminotransferase (AST; r = 0.289), serum aspartate aminotransferase (ALT; r = 0.465), lactate dehydrogenase (LDH; r = 0.206), uric acid (UA; r = 0.402), low-density lipoprotein cholesterol (LDL-C; r = 0.279), and C-reactive protein (CRP; r = 0.160) (all *p* < 0.001). In contrast, FLI was negatively correlated with estimated glomerular filtration rate (eGFR; r = –0.070), total bilirubin (TBIL; r = –0.213), high-density lipoprotein cholesterol (HDL-C; r = –0.459), and 25-hydroxy vitamin D (Vit D; r = –0.081) (all *p* < 0.001).

To estimate FLI while avoiding circularity, we excluded BMI, waist circumference, triglycerides, and γ- GT—the four components embedded in the original FLI formula—from model inputs. Using the remaining variables, we constructed predictive models via both multiple MLR and MARS. Model performance was evaluated using SMAPE, RRSE, and RMSE (Table 5). The MARS model consistently outperformed MLR across all metrics: SMAPE (2.37 vs. 2.46), RRSE (1.24 vs. 1.27), and RMSE (32.04 vs. 32.80), indicating superior predictive accuracy and generalizability.

The final MARS model consisted of 16 basis functions derived from 10 major predictors: age, SBP, WBC, FPG, ALT, AST, UA, HDL-C, LDL-C, and CRP (Table 6). The relative importance of these predictors, as reported in the Abstract and Conclusions, was determined using the standardized nsubsets importance measure from the earth R package, which evaluates the contribution of each variable to the model’s overall fit [29].

**Figure 2:**
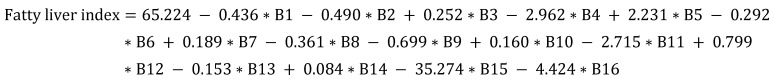
Equation derived from the multiple adaptive regression splines method.

The model equation is illustrated in Figure 2, and a detailed step-by-step implementation procedure for model replication in Microsoft Excel is provided in Table 7. In brief, users can enter individual predictor values (e.g., age, SBP, and laboratory parameters), calculate hinge functions using the syntax MAX(0, x − knot) or MAX(0, knot − x), multiply each term by its corresponding coefficient, and sum all terms with the intercept (65.224) to estimate the FLI.

**Figure 3:**
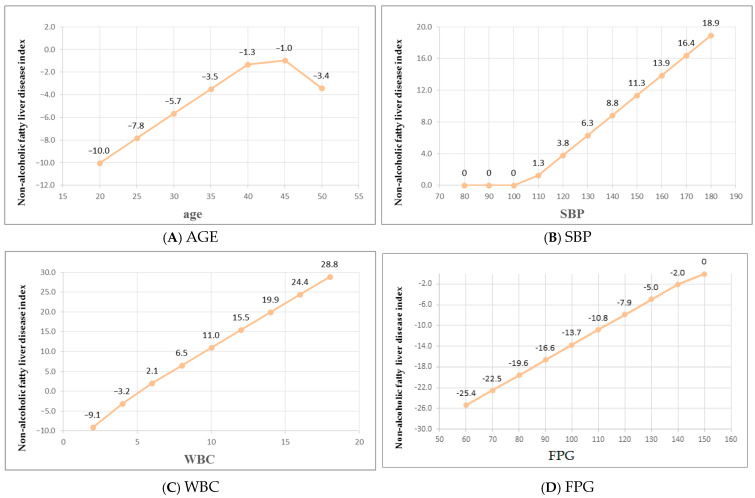

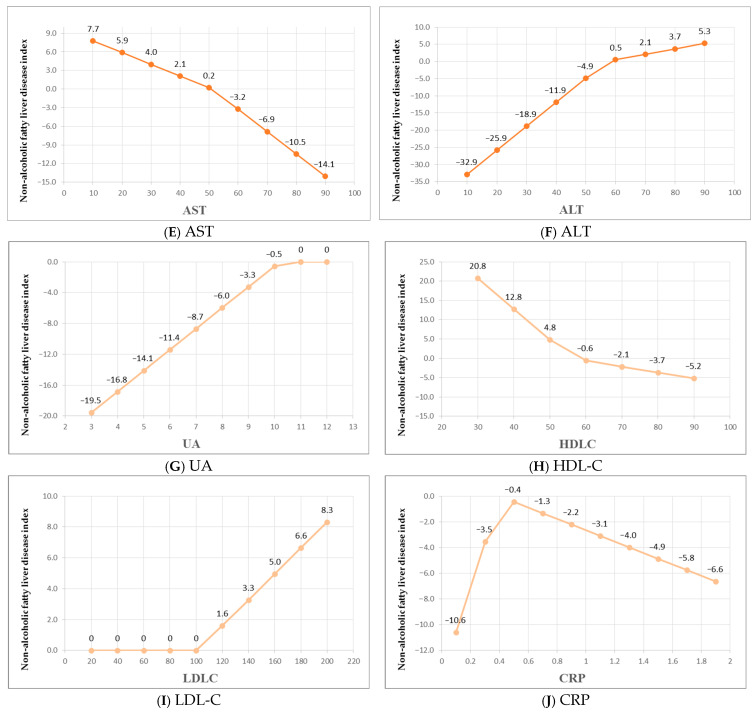
Relationships between different predictors and NAFLD. (**A**) Age; (**B**) SBP; (**C**) WBC; (**D**) FPG; (**E**) AST; (**F**) ALT; (**G**) UA; (**H**) HDLC; (**I**) LDLC; (**J**) CRP. Note: systolic blood pressure (SBP), white blood cell count (WBC), fasting plasma glucose (FPG), aspartate aminotransferase (ALT), alanine aminotransferase (AST), uric acid (UA), high-density lipoprotein cholesterol (HDL-C), low-density lipoprotein cholesterol (LDL-C).

The nonlinear and piecewise linear relationships between individual predictors and FLI are depicted from Figure 3A–J. As shown in Figure 3A, age exhibited a two-phase linear relationship, with a modest increase below 43 years and a steeper slope beyond this threshold. SBP demonstrated a threshold pattern, remaining relatively constant below approximately 105 mmHg and increasing linearly thereafter (Figure 3B). WBC displayed a V-shaped association, with FLI increasing both below and above the knot value of 5.07 ×10^3^/μL (Figure 3C). FPG showed a slight decline in FLI up to 147 mg/dL, followed by a plateau (Figure 3D). For AST, FLI increased up to 51 IU/L and declined thereafter, suggesting a biphasic trend (Figure 3E). ALT exhibited an inverse hinge effect, with a sharp rise in FLI as AST approached 57 IU/L from below, and a milder increase beyond this point (Figure 3F). UA showed a steep elevation in FLI as levels approached 10.2 mg/dL, with little change thereafter (Figure 3G). HDL-C was inversely associated with FLI, which decreased rapidly up to 56 mg/dL and continued to decline gradually at higher concentrations (Figure 3H). LDL-C had no apparent effect below approximately 101 mg/dL but contributed positively to FLI at higher levels (Figure 3I). Finally, CRP demonstrated a strong nonlinear inverse association with FLI, decreasing sharply from 0 to 0.4 mg/dL, and more gradually beyond this range (Figure 3J). While the large negative coefficient for CRP’s left hinge (−35.274) reflects the scaling of this particular hinge function, CRP’s overall importance as the top predictor was confirmed by the standardized nsubsets importance measure.

According to the standardized coefficients, the relative importance of predictors in the final MARS model was ranked as follows: CRP > WBC > UA > HDL-C >ALT > age > AST > FPG > SBP > LDL-C.

## 4. Discussion

Using MARS, we built an equation to estimate FLI and found the most important feature related to MARS for healthy Taiwanese young men. This work represents the first use of MARS in this field [31,32,33,34] and presents the following novel contributions: (1) Using MARS to build an equation. (2) Using FLI rather than binary data (i.e., NAFLD present or not). (3) From the coefficients in the equation, determining the relative importance of these features. (4) Focusing on young healthy men without medication, which might have otherwise impacted the independent variables. We thus consider our findings to be reliable.

Previous studies largely used the presence or absence of NAFLD as the dependent variable (categorical), with results presented as area under receiver operating curve or odds ratio. In contrast, the present study used FLI and machine learning methods. Since the FLI equation used TG, waist ratio, and BMI, we excluded these three variables in the machine learning model to better reveal the deeper pathophysiology of the NAFLD without the confounding effects from body weight.

Based on the coefficients, we discuss the equation features below in order of descending significance.

CRP is found to be the most important factor. Elevated C-reactive protein (CRP) levels, a marker of systemic inflammation, are consistently associated with the presence and progression of non-alcoholic fatty liver disease (NAFLD). Multiple studies have found that higher CRP levels correlate with liver fat accumulation, disease severity, and NAFLD development risk, even after adjusting for confounding factors like obesity [35,36]. Foroughi et al., also found that it is related to the severity of NAFLD [37]. The underlying pathophysiology of their relationship could be explained by the chronic low-grade inflammation driven by cytokines such as interleukin-6, which stimulate CRP production in the liver and visceral fat [37]. CRP is also known to upregulate nuclear factor κ-light-chain-enhancer of activated β cells signaling, a central driver of inflammation, which promotes the release of cytokines [38]. Our result further supports these hypotheses, confirming the role of CRP in NAFLD.

Though CRP and WBC are both markers for inflammation, they have fundamental differences. Increased number of WBC is a response to infection, injury, or other inflammatory stimuli, reflecting the body’s mobilization of immune defenses [39]. As previously noted, CRP participates in the immune response by activating the complement pathway, enhancing phagocytosis, and modulating cytokine production [40]. These differences could support our results and demonstrate that CRP and WBC have independent effects on NAFLD.

UA is the third most important factor contributing to FLI. In a prospective study, Xu et al. demonstrated that higher UA levels were a risk factor for NAFLD in 6890 subjects followed for 3 years [41]. However, the authors treated NAFLD as a binary variable, and thus their results are less than fully persuasive. Other studies also support our finding [42,43]. The mechanisms behind this relationship are hypothesized to the following three causes: (1) lipid metabolism dysregulation; (2) oxidative stress; and (3) fructose metabolism [44,45], but a detailed discussion of these proposed mechanisms is beyond the scope of the present study.

The fourth most important factor was a negative association between HDL-C and FLI, highlighting that increased HDL-C might have a protective role in NAFLD. Xuan et al. pointed out that this relationship could be explained by the role of reverse cholesterol transportation that removes cholesterol from the liver [46], a finding supported by other studies [47,48].

The next key variable is ALT. Of note, AST is also in the equation, which indicates that these two enzymes have independent impacts on FLI. Their differences are clearly explained in Table 8 [49,50] and are compatible with our equation.

Other than ALT (coefficient = 0.699), the following variables had coefficients less than 0.5 compared to that of CRP (35.3).

1. Age:

NAFLD prevalence and risk factors are age-dependent, increasing with age in women (especially around menopause), peaking at middle age in men, and tending to decline in very old age [51,52]. NAFLD is common in the elderly and tends to have a more severe course in older adults, with higher risks of complications like non-alcoholic steatohepatitis (NASH), cirrhosis, hepatocellular carcinoma, and cardiovascular disease.

2. FPG:

The relationship between FPG and NAFLD is characterized by a positive, independent, and nonlinear association [53]. The underlying mechanism is that elevated FPG reflects impaired glucose metabolism and insulin resistance, which promote hepatic fat accumulation [54].

3. SBP:

Similar to age and FPG, the relationship between SBP and NAFLD is bidirectional and involves complex metabolic and inflammatory mechanisms. Zhang et al., and Maeda et al., suggest that NAFLD is associated with higher SBP, DBP, and pulse pressure. This relationship may be mediated by insulin resistance and type 2 diabetes, which are common in NAFLD and contribute to hypertension development [55,56].

4. LDL-C:

The last variable in the equation was LDL-C which has an independent association with increased risk for NAFLD [57]. Zhang et al. reported that patients with NAFLD also had higher LDL-C, TG, and low HDL-C [58]. Excessive LDL-C might increase fat accumulation via mitochondrial dysfunction, activation of Kupffer cells, and promote hepatic fibrosis [59,60,61].

The continuous estimation of FLI via an interpretable MARS model offers several potential advantages for clinical translation. First, it provides a quantitative risk score that can identify individuals in the early or subclinical stages of fatty liver, facilitating earlier intervention. Second, the model highlights specific, modifiable biomarkers (e.g., CRP, UA, HDL-C) as key drivers of FLI, suggesting that interventions targeting systemic inflammation, uric acid metabolism, or lipid profiles may be beneficial even in the absence of overt obesity. Finally, the simplicity of the equation—requiring only routine laboratory and clinical measures—allows for easy integration into electronic health records or health screening platforms to automate FLI estimation and flag at-risk individuals for further evaluation or lifestyle counseling.

Our study is subject to certain limitations. First, its cross-sectional design is less definitive for establishing causality than a longitudinal study. Future longitudinal research would better clarify the importance of these variables for NAFLD development. Second, our study focused exclusively on young Taiwanese men (aged 20–50 years) without alcohol consumption or medications for metabolic conditions. This homogeneous cohort was deliberately selected to minimize confounding and to clearly identify early risk factors, but it necessarily limits the immediate generalizability of our predictive equation to women, older adults, other ethnic groups, or individuals with treated comorbidities or alcohol use. Future validation studies should include female participants, given the well-documented sex differences in NAFLD epidemiology and pathophysiology. The equation should therefore be viewed as specifically developed for and validated in this demographic. Future studies are needed to validate and potentially adapt this model to more diverse populations, including women, multi-ethnic cohorts, and individuals across a broader age range and health status spectrum. Third, several tables and figures referenced in the text (Table 1, Table 2, Table 3, Table 4, Table 5, Table 6, Table 7 and Table 8, Figure 1, Figure 2 and Figure 3) are not included in this manuscript version but would be essential for full interpretation of the results in a published article. The absence of these visual aids limits the reader’s ability to fully appreciate the relationships described.

## 5. Conclusions

Using MLR as a benchmark for comparison, the present study finds that MARS outperformed traditional MLR. By using MARS, an equation was built with r2 equals to 0.627. According to the coefficient, CRP is the most important feature, followed by WBC, UA, HDL-C, ALT, age, AST, FPG, SBP, and LDL in healthy young Taiwanese men. This equation is specifically tailored to the demographic characteristics of our study population (young, medication-free Taiwanese men) and requires validation in broader populations before wider clinical application.

## Figures and Tables

**Table 1:** Variable unit and description.

Variables	Unit and Description
Age	Years
Marriage status	(1) Unmarried (2) Married
Education (Edu.)	(1) Illiterate (2) Elementary school (3) Junior high school (4) High school (vocational) (5) Junior college (6) University (7) Graduate school or above
Income	NTD/year (1) Below $200,000 (2) $200,001–$400,000 (3) $400,001–$800,000 (4) $800,001–$1,200,000 (5) $1,200,001–$1,600,000 (6) $1,600,001–$2,000,000 (7) More than $2,000,000
Systolic blood pressure (SBP)	mmHg
Diastolic blood pressure (DBP)	mmHg
White blood cell (WBC)	×10^3^/μL
Hemoglobin (Hb)	×10^6^/μL
Platelets (Plt)	×10^3^/μL
Fasting plasma glucose (FPG)	mg/dL
Total bilirubin (TBIL)	mg/dL
Albumin (Alb)	mg/dL
Globulin (Glo)	g/dL
Alkaline Phosphatase (ALP)	IU/L
Alanine aminotransferase (AST)	IU/L
Aspartate aminotransferase (ALT)	IU/L
Lactate dehydrogenase (LDH)	mg/dL
Estimated glomerular filtration rate (eGFR)	mg/dL
Uric acid (UA)	mg/dL
Triglycerides (TG)	mg/dL
High density lipoprotein cholesterol (HDL-C)	mg/dL
Low density lipoprotein cholesterol (LDL-C)	mg/dL
Plasma calcium concentration (Ca)	mg/dL
Plasma phosphate concentration (P)	mg/dL
Thyroid stimulating hormone (TSH)	μIU/mL
C-reactive protein (CRP)	mg/dL
25-OH Vitamin D (Vit D)	ng/mL
Non-alcoholic fatty liver disease index (NAFLD)	$\frac{{(e}^{\left(0.953\times \ln\left(TG\right)+0.139\times BMI+0.718\times \ln\left(\gamma -GT\right)+0.053\times WC-15.745\right)})}{\left(1+e^{\left(0.953\times \ln\left(TG\right)+0.139\times BMI+0.718\times \ln\left(\gamma -GT\right)+0.053\times WC-15.745\right)}\right)}\times 100$
Smoking area	—
Betel nut area	—
Sport area	—
Sleeping hours	(1) 0~4 h (2) 4~6 h (3) 6~8 h (4) more than 8 h

**Table 2:** Performance metrics for evaluating the prediction accuracy of the proposed model.

Metrics	Description	Calculation
MAPE	Mean Absolute Percentage Error	$MAPE=\frac{1}{n}\sum_{i=1}^{n}\left|\frac{y_{i}-{\hat{y}}_{i}}{y_{i}}\right|\times 100$
RRSE	Root Relative Squared Error	$RRSE=\sqrt{\frac{\sum_{i=1}^{n}{\left(y_{i}-{\hat{y}}_{i}\right)}^{2}}{\sum_{i=1}^{n}{\left(y_{i}-\bar{Y}\right)}^{2}}}$
RMSE	Root Mean Squared Error	$RMSE=\sqrt{\frac{1}{n}\sum_{i=1}^{n}{\left(y_{i}-{\hat{y}}_{i}\right)}^{2}}$

where ${\hat{y}}_{i}$ and $y_{i}$ respectively represent predicted and actual values, and n stands for the number of instances.

**Table 3:** Participant demographics and the testing conditions for non-alcoholic fatty liver disease index and various subgroup variables.

Variables	Mean ± SD	
Age	36.80 ± 7.77	
Systolic blood pressure (SBP)	121.34 ± 14.07	
Diastolic blood pressure (DBP)	78.65 ± 10.43	
White blood cell (WBC)	6.23 ± 1.57	
Hemoglobin (Hb)	15.41 ± 1.08	
Platelets (Plt)	232.86 ± 50.35	
Fasting plasma glucose (FPG)	100.20 ± 16.44	
Total bilirubin (TBIL)	1.04 ± 0.41	
Albumin (Alb)	4.51 ± 0.21	
Globulin (Glo)	3.04 ± 0.32	
Alkaline phosphatase (ALP)	61.40 ± 17.68	
Alanine aminotransferase (AST)	25.44 ± 13.12	
Aspartate aminotransferase (ALT)	36.70 ± 29.98	
Lactate dehydrogenase (LDH)	161.27 ± 33.11	
Estimated glomerular filtration rate (eGFR)	84.92 ± 11.84	
Uric acid (UA)	6.58 ± 1.32	
Triglycerides (TG)	125.92 ± 105.76	
High density lipoprotein cholesterol (HDL-C)	51.49 ± 11.18	
Low density lipoprotein cholesterol (LDL-C)	125.10 ± 33.51	
Plasma calcium concentration (Ca)	9.73 ± 0.36	
Plasma phosphate concentration (P)	3.69 ± 0.50	
Thyroid stimulating hormone (TSH)	1.54 ± 2.32	
C-reactive protein (CRP)	0.22 ± 0.38	
25-OH Vitamin D (Vit D)	23.08 ± 7.62	
Non-alcoholic fatty liver disease (NAFLD) index	32.57 ± 26.73	
**Ordinal variables**		
Marriage status	n (%)	*p*-value
Unmarried	2236 (43.2)	<0.001 ***
Married	2939 (56.8)	
Income	n (%)	*p*-value
Below $200,000	335 (6.7)	<0.001 ***
$200,001–$400,000	523 (10.4)	
$400,001–$800,000	1792 (35.6)	
$800,001–$1,200,000	1312 (26.1)	
$1,200,001–$1,600,000	470 (9.3)	
$1,600,001–$2,000,000	240 (4.8)	
More than $2,000,000	357 (7.1)	
Education	n (%)	*p*-value
Elementary school	1 (0.01)	
Junior high school	35 (0.7)	
High school (vocational)	587 (11.5)	<0.001 ***
Junior college	538 (10.5)	
University	2591 (50.6)	
Graduate school or above	1366 (26.7)	
Sleep hours	n (%)	*p*-value
0–4 h/day	37 (0.7)	0.001 ***
4–6 h/day	1443 (27.3)	
6–7 h/day	2716 (51.4)	
7–8 h/day	941 (17.8)	
8–9 h/day	124 (2.3)	
>9 h/day	24 (0.5)	

*** *p* < 0.001.

**Table 4:** The r values of Pearson correlation between NAFLD index and demographic biochemistry.

Age	SBP	DBP	WBC	Hb	Pla	FPG	TBIL	Alb	Glo	ALP	AST
0.172 **	0.344 ***	0.344 ***	0.354 ***	0.193 ***	0.205 ***	0.278 ***	−0.213 ***	−0.011	0.171 ***	0.156 ***	0.289 ***
**ALT**	**LDH**	**eGFR**	**UA**	**HDL-C**	**LDL-C**	**Ca**	**P**	**TSH**	**CRP**	**Vit D**	
0.465 ***	0.206 ***	−0.070 ***	0.402 ***	−0.459 ***	0.279 ***	0.010	−0.017	0.038 **	0.160 ***	−0.081 ***	

NAFLD: Non-alcoholic fatty liver disease; SBP: systolic blood pressure; DBP: diastolic blood pressure; WBC: leukocyte; Hb: hemoglobin; Pla: platelets; FPG: fasting plasma glucose; TBIL: total bilirubin; Alb: albumin; Glo: globulin; ALP: alkaline phosphatase; ALT: aspartate aminotransferase; AST: alanine aminotransferase; LDH: lactate dehydrogenase; eGFR: estimated glomerular filtration rate; UA: uric acid; HDL-C: high-density lipoprotein cholesterol; LDL-C: low-density lipoprotein cholesterol; CRP: C-reactive protein; Vit D: vitamin D. ** *p* < 0.01, *** *p* < 0.001.

**Table 5:** Average performance of linear regression and multivariate adaptive regression splines.

	SMAPE	RRSE	RMSE
MARS	2.3676	1.2433	32.0395
MLR	2.4566	1.273	32.8048

MLR: Multiple linear regression, MARS: multivariate adaptive regression splines. SMAPE: Symmetric Mean Absolute Percentage Error, RRSE: Root Relative Squared Error, RMSE: Root Mean Squared Error.

**Table 6:** List of basis functions Bi of the MARS model and their coefficients, ai.

	Definition	α1
Intercept	—	65.224
B 1	max (43-age)	−0.436
B 2	max (age-43)	−0.490
B 3	max (SBP-105)	0.252
B 4	max (5.07-WBC)	−2.962
B 5	max (WBC-5.07)	2.231
B 6	max (147-FPG)	−0.292
B 7	max (51-AST)	0.189
B 8	max (AST-51)	−0.361
B 9	max (57-ALT)	−0.699
B 10	max (ALT-57)	0.160
B 11	max (10.2-UA)	−2.715
B 12	max (56-HDLC)	0.799
B 13	max (HDLC-56)	−0.153
B 14	max (LDLC-101)	0.084
B 15	max (0.4-CRP)	−35.274
B 16	max (CRP-0.4)	−4.424

SBP: Systolic blood pressure; WBC: leukocyte; FPG: fasting plasma glucose; AST: alanine aminotransferase; ALT: aspartate aminotransferase; UA: uric acid; HDL-C: high-density lipoprotein cholesterol; LDL-C: low-density lipoprotein cholesterol; CRP: C-reactive protein.

**Table 7:** The functions of our equation in Excel.

	A	B	C
1	Type Age	=MAX (0, 43-A1)	=−0.436 × B1
2		=MAX (0, A1-43)	=−0.490 × B2
3	Type SBP	=MAX (0, A3-105)	=0.252 × B3
4	Type WBC	=MAX (0, 5.07-A4)	=−2.962 × B4
5		=MAX (0, A4-5.07)	=2.231 × B5
6	Type FBG	=MAX (0, 147-A6)	=−0.292 × B6
7	Type AST	=MAX (0, 51-A7)	=0.189 × B7
8		=MAX (0, A7-51)	=−0.361 × B8
9	Type ALT	=MAX (0, 57-A9)	=−0.699 × B9
10		=MAX (0, A9-57)	=0.160 × B10
11	Type UA	=MAX (0, 10.2-A11)	=−2.715 × B11
12	Type HDLC	=MAX (0, 56-A12)	=0.799 × B12
13		=MAX (0, A12-56)	=−0.153 × B13
14	Type LDLC	=MAX (0, A14-101)	=0.084 × B14
15	Type CRP	=MAX (0, 0.4-A15)	=−35.274 × B15
16		=MAX (0, A15-0.4)	=−4.424 × B16
17			
18	NAFLD		
19	=65.224 + SUM(C1:C16)		

**Table 8:** Differences between ALT and AST.

Aspect	ALT	AST
Primary function	Alanine-pyruvate transamination	Glutamate-oxaloacetate transamination; malate-aspartate shuttle
Tissue specificity	More liver-specific	Present in liver and other tissues
Role in NAFLD	Marker of hepatocyte injury	Promotes lipotoxicity and mitochondrial oxidative stress
Impact on liver metabolism	Reflects liver cell damage	Enhances CAC anaplerosis and ROS production, driving hepatocyte apoptosis
Association with metabolic syndrome	Less direct	Positively associated with hypertension and lipid abnormalities

## Data Availability

Data available on request due to privacy/ethical restrictions.
